# Identifying target ion channel-related genes to construct a diagnosis model for insulinoma

**DOI:** 10.3389/fgene.2023.1181307

**Published:** 2023-09-12

**Authors:** Shuangyang Mo, Yingwei Wang, Wenhong Wu, Huaying Zhao, Haixing Jiang, Shanyu Qin

**Affiliations:** ^1^ Gastroenterology Department, Liuzhou People’s Hospital Affiliated to Guangxi Medical University, Liuzhou, China; ^2^ Gastroenterology Department, The First Affiliated Hospital of Guangxi Medical University, Nanning, China

**Keywords:** insulinoma, ion channel, machine learning, diagnosis model, immune infiltration

## Abstract

**Background:** Insulinoma is the most common functional pancreatic neuroendocrine tumor (PNET) with abnormal insulin hypersecretion. The etiopathogenesis of insulinoma remains indefinable. Based on multiple bioinformatics methods and machine learning algorithms, this study proposed exploring the molecular mechanism from ion channel-related genes to establish a genetic diagnosis model for insulinoma.

**Methods:** The mRNA expression profile dataset of GSE73338 was applied to the analysis, which contains 17 insulinoma samples, 63 nonfunctional PNET (NFPNET) samples, and four normal islet samples. Differently expressed ion channel-related genes (DEICRGs) enrichment analyses were performed. We utilized the protein–protein interaction (PPI) analysis and machine learning of LASSO and support vector machine-recursive feature elimination (SVM-RFE) to identify the target genes. Based on these target genes, a nomogram diagnostic model was constructed and verified by a receiver operating characteristic (ROC) curve. Moreover, immune infiltration analysis, single-gene gene set enrichment analysis (GSEA), and gene set variation analysis (GSVA) were executed. Finally, a drug–gene interaction network was constructed.

**Results:** We identified 29 DEICRGs, and enrichment analyses indicated they were primarily enriched in ion transport, cellular ion homeostasis, pancreatic secretion, and lysosome. Moreover, the PPI network and machine learning recognized three target genes (*MCOLN1*, *ATP6V0E1*, and *ATP4A*). Based on these target genes, we constructed an efficiently predictable diagnosis model for identifying insulinomas with a nomogram and validated it with the ROC curve (AUC = 0.801, 95% CI 0.674–0.898). Then, single-gene GSEA analysis revealed that these target genes had a significantly positive correlation with insulin secretion and lysosome. In contrast, the TGF-beta signaling pathway was negatively associated with them. Furthermore, statistically significant discrepancies in immune infiltration were revealed.

**Conclusion:** We identified three ion channel-related genes and constructed an efficiently predictable diagnosis model to offer a novel approach for diagnosing insulinoma.

## Introduction

Pancreatic neuroendocrine tumors (PNETs) are rare neoplasms derived from intrapancreatic endocrine cells, comprising 2%–10% of all pancreatic tumors. PNETs are heterogeneous tumors and can be generally classified as functional and nonfunctional, depending on the differentiation of secreting hormones or not (Chen et al., 2020). Insulinoma (islet β-cell origin) is the most common subtype of functional PNET, leading to recurrent hypoglycemia due to unadjustable endogenous hyperinsulinism. Abnormal insulin hypersecretion is a necessary condition of insulinoma diagnosis. Although the benign form of insulinoma is common and malignant insulinoma only account for approximately 6%–10% of all insulinoma (Liu et al., 2018), the deferred or erroneous diagnosis of hypoglycemia and other customary symptoms usually raises the seriousness and mortality of insulinoma (Yang et al., 2015). Actually, patients with insulinoma are often misdiagnosed for long periods (Imamura, 2010). Indeed, the accurate diagnosis of insulinoma is a significant challenge based on the varied clinical presentations, nonspecific biochemical tests, and low-specificity clinical diagnostic model (Giannis et al., 2020), and it is also difficult to identify insulinoma in an early stage from nonfunctional PNET (NFPNET) (Karakose et al., 2020). Nonetheless, although we have made many advances in the awareness of insulinoma with their hyperinsulinism and clinical symptoms, the molecular mechanisms of regulating these processes and the genetic diagnostic model remain unclear.

The synthesis, storage, and secretion of insulin from islet β-cells are modulated by complicated mechanisms, including endocrine, paracrine, and complex signaling pathways (Pisani et al., 2016). The ion channel widely distributes in biological membranes and plays a vital role in tightly regulating intracellular homeostasis, hormone secretion, and signal transduction. The anomalous modulation of ion channels can result in diverse diseases, including diabetes and cancers (Singh et al., 2018; Jacobson and Shyng, 2020). Insulin secretion follows ATP production, membrane depolarization, and ultimately opening of voltage-gated calcium ion channels. This is the classical signaling pathway in the modulation of the secretion of insulin (Volta et al., 2019). The islet β-cells can perceive the ascendance of serum glucose and trigger the cellular glucose uptake, leading to an increase in producing ATP and other metabolites. The increase in ATP promotes voltage-gated calcium ion activation and exocytosis of insulin-containing granules. T. Stuhlmann reported that VRAC, a volume-regulated anion channel, can promote insulin secretion by regulating the depolarization of islet β-cells to respond to glucose induction (Stuhlmann et al., 2018).

Furthermore, the increasing glucose evokes an anionic flux by stimulating a volume-sensitive chloride channel of the islet *β*-cells (Li et al., 2007). Diazoxide, an agonist of ATP-sensitive potassium channels (KATP), can suppress insulin secretion and reverse hypoglycemia in patients (Barrosse-Antle et al., 2017). It was reported that some non-steroidal anti-inflammatory drugs (NSAIDs) might cause adverse reactions of hypoglycemia by disturbing varied ion channel functions in islet *β*-cells (Li et al., 2007). The sympathetic and autonomic parasympathetic fibers also regulate the secretion function of islet β-cells, while ion channels are pivotal in mediating the signal transmission of neural synapses (Alvarsson et al., 2020).

Together, it is indicated that the ion channels might play a central regulatory intermediary and executor in multifarious insulin secretion signals. The regulation of various ion channel activities effectively influences the excitability and insulin secretion of islet β-cells (Park et al., 2014). In contrast, the activity of particular ion channels and intracellular ion concentrations is also modulated by insulin (Peng et al., 2022). Although insulinoma and NFPNET arise from intrapancreatic islet cells, they show extreme heterogeneity (Quevedo et al., 2020). Along these lines, we hypothesized that the heterogeneity of ion channels might involve uncontrollable insulin secretion of insulinoma compared with NFPNET. It might provide a novel insight and a therapeutical target for insulinoma in the future. However, our understanding of the specific molecule characteristic and difference of ion channels within insulinoma and NFPNET is limited. Hence, a novel diagnostic model based on molecular mechanisms, especially the ion channel-related genes, is needed.

Nowadays, along with the advancement of high-throughput sequencing and microarray technologies, bioinformatics has played a vital role in life science research, which was utilized to analyze the differentially expressed mRNA and predict the potential therapeutic targets in a particular disease. Bioinformatic analysis is an efficacious approach to discovering biomarkers and etiopathogenesis of diseases, and it could provide an estimable foundation for further studies (Peng et al., 2022). Therefore, in this research, we analyzed the Gene Expression Omnibus (GEO) (Clough and Barrett, 2016) dataset with bioinformatic methods and machine learning to identify the target ion channel-related genes among insulinoma and NFPNET groups. Furthermore, a predictive diagnostic model was established and evaluated.

## Materials and methods

### Data source of microarray

**FIGURE 1:**
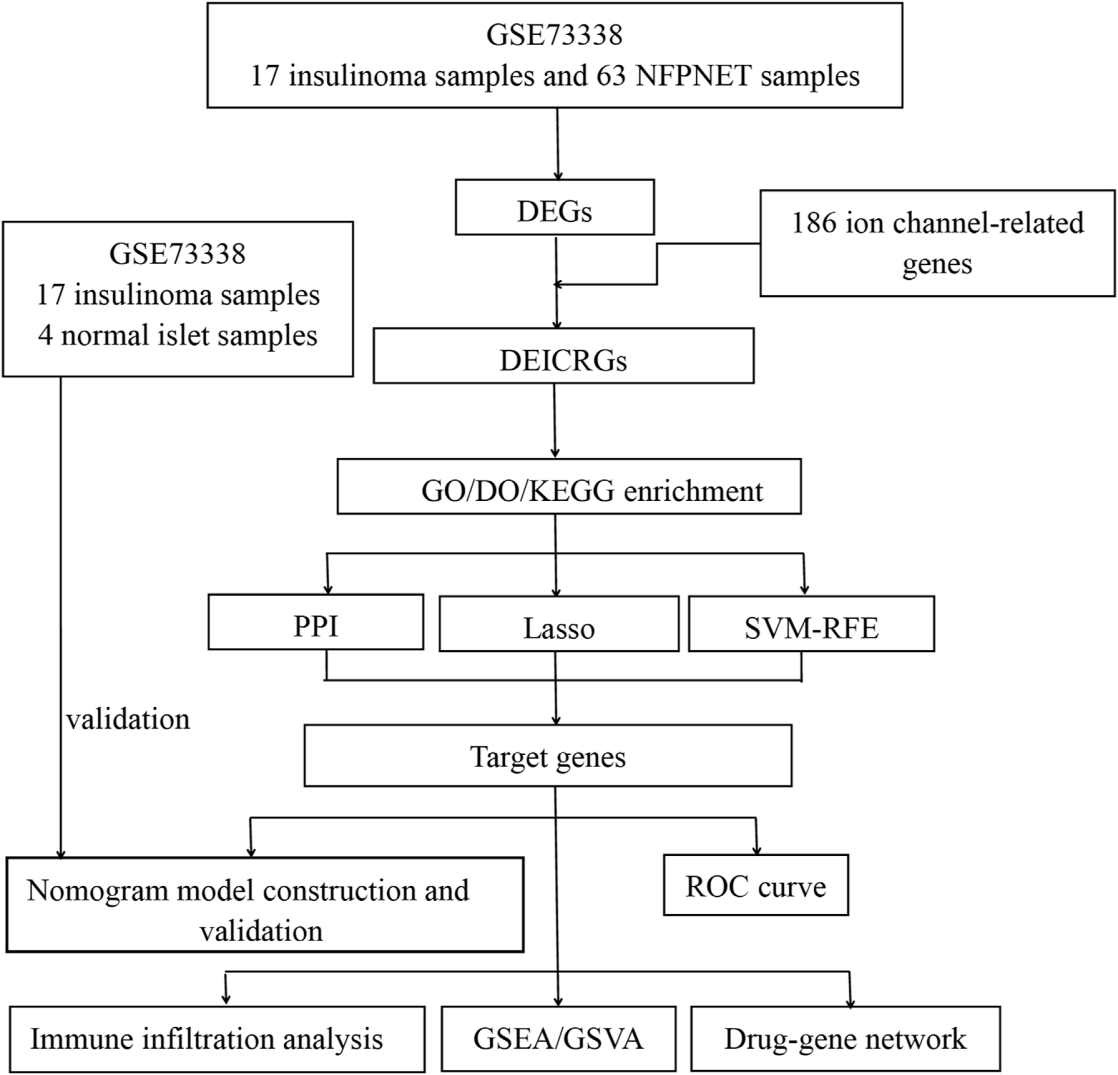
Flowchart of the study.

The procedure of analysis for this research is shown in Figure 1. The inclusion criteria were set as the test specimens included should be derived from humans, and these independent expression profiles contain the largest sample size. GSE73338 was enrolled in this study, which was downloaded from the GEO database. There were 63 NFPNET, 17 insulinoma, and four normal islet samples’ mRNA expression profiling in GSE73338. Then, they were divided into a training set (NFPNET and insulinoma) and an internal validation set (insulinoma and normal islet) because NFPNET and insulinoma samples contain the largest sample size. Additional details are provided in Table 1.

**TABLE 1:** Details of the GEO data.

Dataset	Platform	Number of samples (insulinoma/NFPNET/normal islet)
GSE73338	GPL20945 18.5K human oligo microarrays obtained from the Ohio State University Cancer Center	84 (17/63/4)

GEO, Gene Expression Omnibus.

### Identifying differently expressed ion channel-related genes

The differently expressed genes (DEGs) between the insulinoma and NFPNET samples were identified from normalized and preprocessed data using the GEO2R tool (Barrett et al., 2013). The screening threshold was stated at |log2 Fold Change| >0.585 and *p* < 0.05. The 186 genes participating in the ion channel pathway were downloaded from the GeneCards database (https://www.genecards.org/). The R limma package was utilized to identify the differently expressed ion channel-related genes (DEICRGs) with the threshold as *p* < 0.05.

### Enrichment analyses of GO, DO, and KEGG

Gene Ontology (GO) enrichment [including biological process (BP), cellular component (CC), and molecular function (MF)] analysis and Kyoto Encyclopedia of Genes and Genomes (KEGG) pathway analysis were applied by utilizing the R clusterProfiler package (Yu et al., 2012). The false discovery rate (FDR) was calculated via Benjamini–Hochberg (BH) adjustment. The cutoff criterion was q-value <0.05. We utilized the R DOSE package to apply the enrichment analysis of disease ontology (DO) terms (Yu et al., 2015). Finally, the R ggplot2 and pathview packages were utilized to visualize the significant results of these enrichment analyses.

### Machine learning of lasso and support vector machine-recursive feature elimination

Ulteriorly, machine learning, such as the lasso regression and support vector machine-recursive feature elimination (SVM-RFE) algorithm, was used to screen the feature genes from DEICRGs. Lasso regression and the optimal parameter λ were determined through 10-fold cross-validation via the R glmnet package with “family = binomial, measure = deviance” and with all other parameters arranged to default (Engebretsen and Bohlin, 2019). Meanwhile, the SVM-RFE method, an effective feature selection technique, was also implemented for distinguishing feature genes from DEICRGs with the R e1071 package (Jiang et al., 2020). Our study identified the best feature genes via the SVM-RFE algorithm based on a maximum 5×CV accuracy and a minimum 5×CV error value simultaneously.

### Protein–protein interaction network and hub gene analyses

The protein–protein interaction network (PPI) was predicted and constructed on the biological database of STRING (https://string-db.org/). We uploaded the DEICRGs to the STRING database to build a PPI network and utilized Cytoscape 3.9.1 software to visualize and further analyze it. The hub genes were identified via the cytoHubba plugin.

### Recognition of target genes

We defined the uniformly present genes in the hub gene set and feature gene set from two machine learning models described previously as target genes.

### Construction of a diagnostic model and evaluation of the diagnostic efficiency

We constructed a diagnostic model with a nomogram based on the target genes by utilizing the R rms package. The calibration curve was established to assess the calibration of the nomogram model by mean absolute error and 1,000 bootstrap samples using the R CalibrationCurves package. Decision curve analysis (DCA) was performed to evaluate the value of net benefits in the nomogram model at the different high-risk thresholds. Finally, whether the nomogram model had favorable predictive effects was evaluated by the clinical impact curve (CIC). Then, a receiver operating characteristic (ROC) curve was applied to further assess the diagnostic efficacy of the model in distinguishing insulinoma from NFPNET via the R glmnet and pROC packages. Additionally, we applied the insulinoma samples and normal islet samples from GSE73338 as the validation set to verify the efficacy of the diagnostic model.

### Immune infiltration analysis

The deconvolution algorithm of CIBERSORT (Newman et al., 2015), which can assess the percentage of 22 infiltrating immune cell subtypes, was used to calculate the immune infiltration of insulinoma and NFPNET tissues via the CIBERSORT R script v1.03. Then, the correlation between each subtype of immune cells and target genes was estimated with Pearson’s correlation analysis and visualized. Furthermore, we obtained 28 immune-related cell gene sets and utilized the single-sample gene set enrichment analysis (ssGSEA) via the R GSVA package to explore the different infiltration enrichment scores of each immune cell subtype in each sample (Barbie et al., 2009; Charoentong et al., 2017). The R limma package was applied to analyze the different infiltration enrichment scores between insulinoma and NFPNET groups. Finally, the results of ssGSEA were visualized with a heatmap and boxplot.

### Gene set enrichment analysis and gene set variation analysis

Following this, we focus on elucidating the potential roles of target genes in insulinoma. A single-gene gene set enrichment analysis (GSEA) for each target gene was performed separately via the R clusterProfiler package. First, all samples were split into the low-expression and high-expression groups according to the expression level of each specific single target gene. Then, GSEA was performed to estimate the significantly different pathways of KEGG within these two groups. Gene set variation analysis (GSVA) is a nonparametric unsupervised method. It was performed to demonstrate the differential enrichment of KEGG pathways and GO terms between these groups similarly. In this study, the R GSVA package was utilized with the gene sets of c2. cp.kegg.symbols.gmt and c5. go.symbols.gmt. They were downloaded from the official site. The threshold standard for statistically significant terms was set as *p* < 0.05.

### Drug–gene interaction network and visualization

We utilized the DGIdb (Edwards et al., 2011) and DrugBank (Wishart et al., 2018) databases to predict existing or/and possibly related drug substances for investigating the drug–gene connection. Furthermore, the data visualization of the drug–gene interaction network was constructed with Cytoscape software.

## Results

### Recognition of DEGs

**FIGURE 2:**
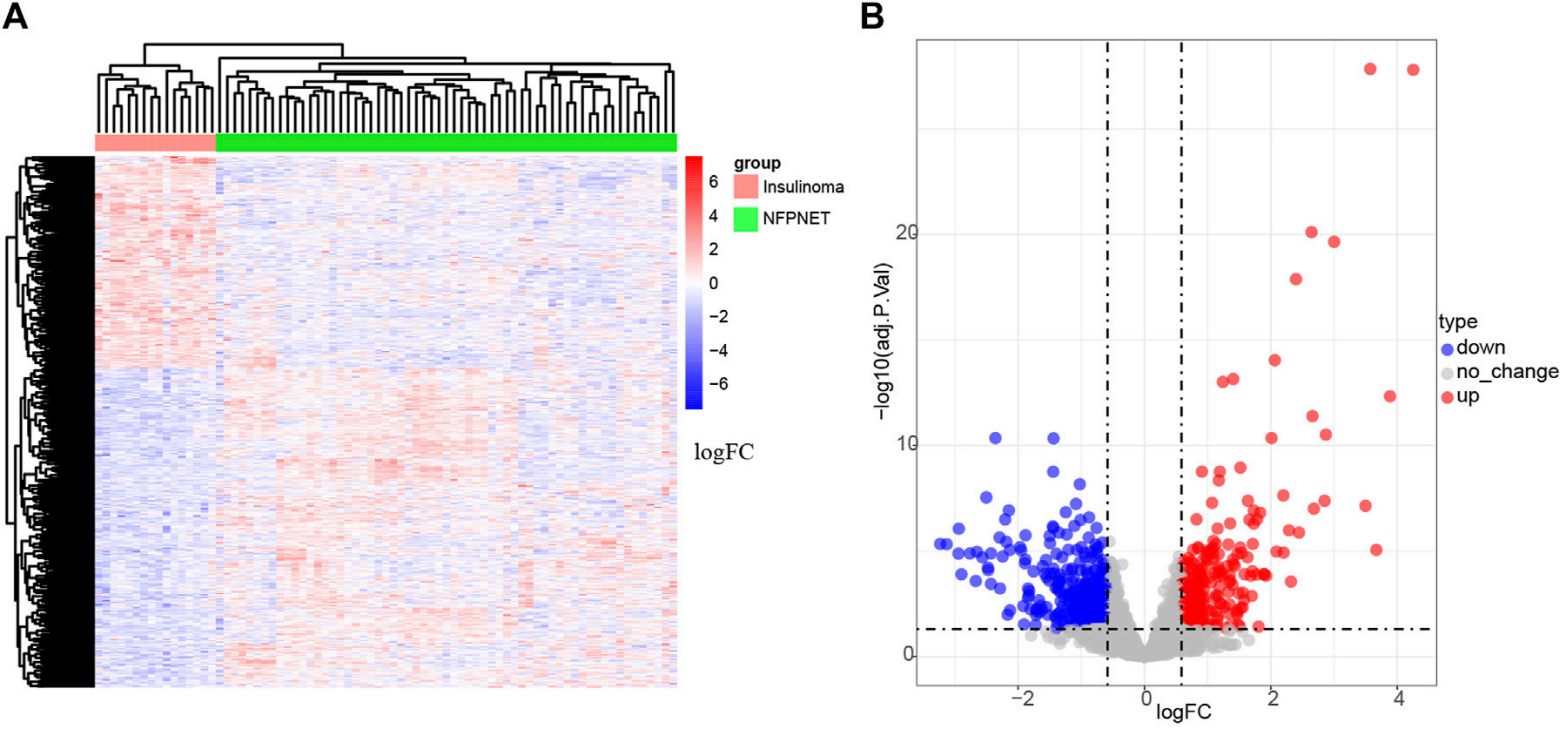
Identification of DEGs. **(A)** Heatmap of DEGs; **(B)** volcano plot of GSE73338.

The mRNA expression profile dataset GSE73338 was normalized. Then, 650 DEGs (containing 259 up-expressed DEGs and 391 down-expressed DEGs) were identified from the GSE73338 dataset, and a volcano plot and heatmap are shown in Figures 2A,B.

### Enrichment analyses of the DEGs

**FIGURE 3:**
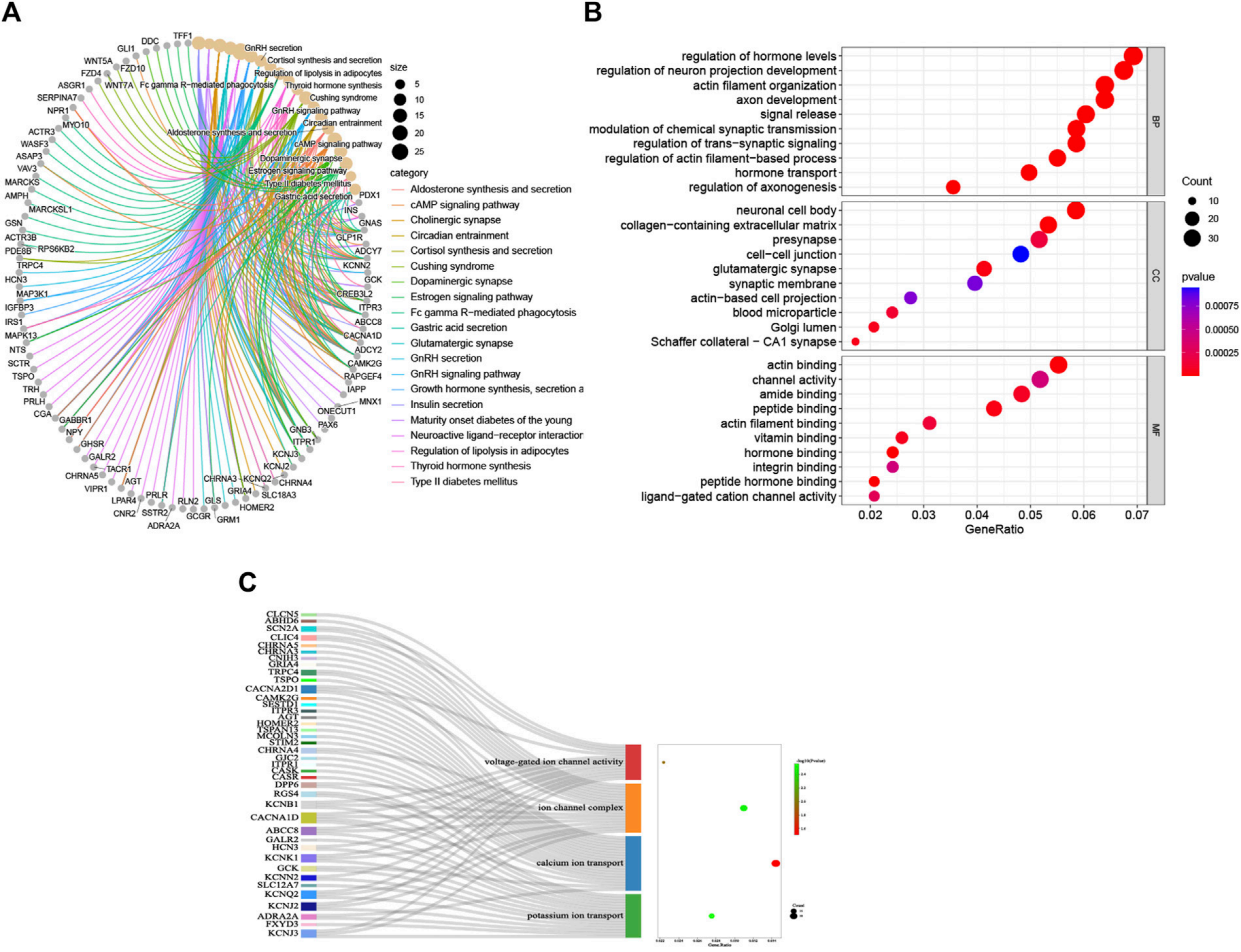
Enrichment analyses of DEGs. **(A)** KEGG signaling pathway; **(B)** GO BP, GO CC, and GO MF; **(C)** details of GO terms (voltage-gated ion channel activity GO term, ion channel complex GO term, calcium ion transport GO term, and potassium ion transport GO term.

The KEGG and GO analyses of the DEGs were executed to indicate their potential biology functions. As displayed in the KEGG cluster (Figure 3A), the DEGs mainly participate in regulating the synthesis, secretion, and action of various endocrine hormones, such as aldosterone, cortisol, thyroid, and growth hormone. In particular, the DEGs are directly involved in managing insulin secretion and the cAMP signaling pathway, which is proven to promote insulin secretion from islet β-cells activated by glucagon. In the GO category, most of the DEGs are located in the synaptic membrane, cell–cell junction, and Golgi lumen, which specifically participate in the regulation of hormone levels, hormone transport, modulation of chemical synaptic transmission, signal release, channel activity, and hormone binding (Figure 3B). Furthermore, we found that many DEGs are closely associated with potassium ion transport, calcium ion transport, ion channel complex, and voltage-gated ion channel activity (Figure 3C). This indicates that the ion channel activity might play a crucial role in modulating insulin secretion from insulinoma.

### Recognition of DEICRGs of insulinoma

**FIGURE 4:**
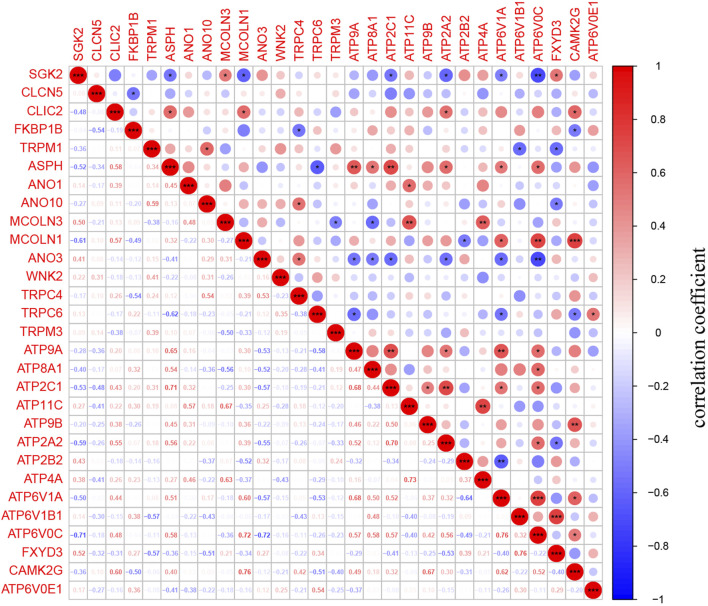
Identification of DEICRGs and correlation matrix. The correlation matrix of DEICRGs. (* means *p* < 0.05; ** means *p* < 0.01; *** means *p* < 0.001).

The 186 genes that participate in the ion channel pathway are presented in Supplementary Material. A total of 29 DEICRGs were identified from the GSE73338 dataset, including 14 up-expressed DEICRGs and 15 down-expressed DEICRGs (Table 2). Then, the heatmap and correlation coefficient diagram of DEICRGs are shown in Supplementary Figure S1 and Figure 4.

**TABLE 2:** DEICRGs of GSE73338.

Regulation	DEICRG
Upregulated (*n* = 14)	TRPM1、ASPH、ANO10、MCOLN1、WNK2、TRPC6、TRPM3、ATP8A1、ATP2C1、ATP4A、ATP6V1A、ATP6V1B1、ATP6V0C、and ATP6V0E1
Downregulated (*n* = 15)	SGK2、CLCN5、CLIC2、FKBP1B、ANO1、MCOLN3、ANO3、TRPC4、ATP9A、ATP11C、ATP9B、ATP2A2、ATP2B2、FXYD3、 and CAMK2G

DEICRGs, differently expressed ion channel-related genes.

### Function enrichment analyses of the DEICRGs

**FIGURE 5:**
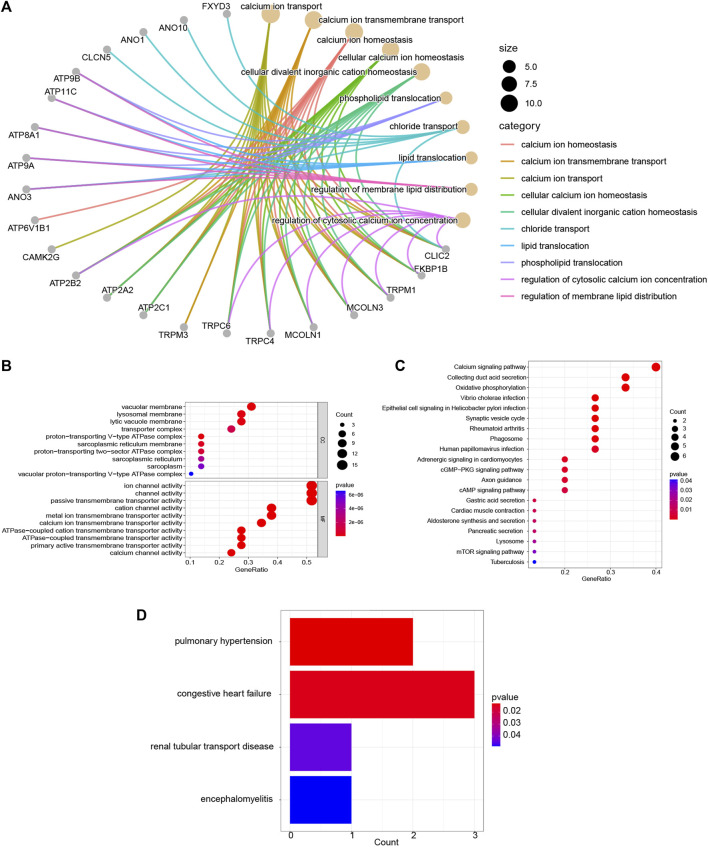
Enrichment analyses of DEICRGs. **(A)** GO BP; **(B)** GP CC and GO MF; **(C)** KEGG pathway; **(D)** DO enrichment.

The biology functions of DEICRGs were performed in GO, KEGG, and DO analyses. Expectedly, the DEICRGs were mainly located in the biological membrane and played the role of the transporter of various inorganic ions across the plasma membrane by regulating the ion channel activity, which contributed to maintaining the cellular metal ion homeostasis (Figures 5A, B). The enrichment analysis results via KEGG pathways show that the DEICRGs are mostly associated with the calcium signaling pathway, oxidative phosphorylation, synaptic vesicle cycle, cGMP−PKG signaling pathway, cAMP signaling pathway, gastric acid secretion, pancreatic secretion, lysosome, and mTOR signaling pathway (Figure 5C). The R DOSE package was applied to investigate the function of DEICRGs further. The results of DO enrichment revealed that DEICRGs might participate in pulmonary hypertension, congestive heart failure, and renal tubular transport disease. These results suggest that the primary functions of DEICRGs may relate to the regulation of ion channel activity, pancreatic secretion, lysosome, and cell signal transmission.

### The machine learning algorithm of lasso and SVM-RFE

**FIGURE 6:**
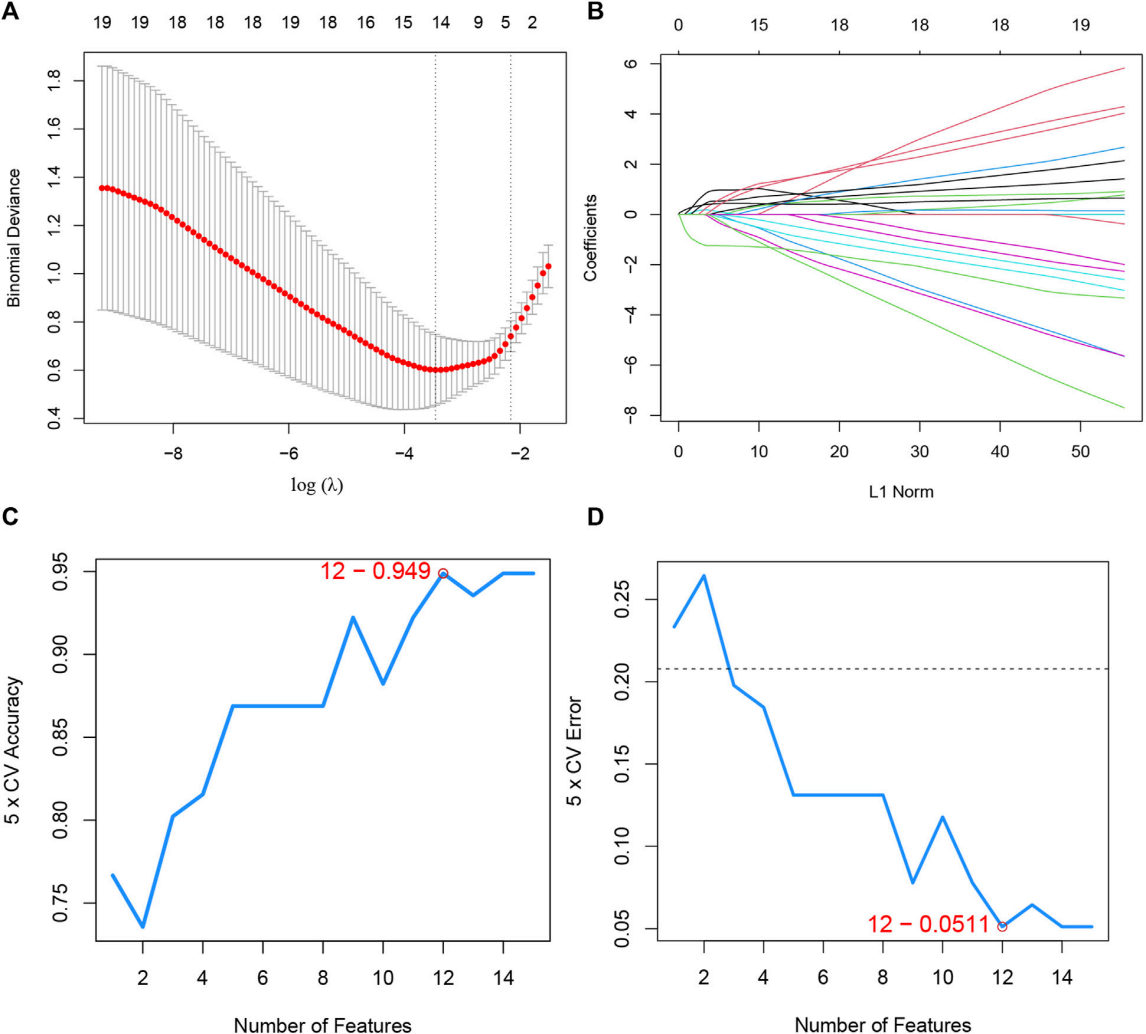
Machine learning. **(A)** the log(*λ*) value was optimally selected by 10-fold cross-validation and plotted by the partial likelihood deviance; **(B)** Processes of lasso regression for identifying variables and mapping each variable to a curve; **(C)** the accuracy (5× CV) is highest as 0.949 when the number of feature genes is 12; **(D)** the error (5× CV) is lowest as 0.051 when the number of feature genes is 12.

Furthermore, to screen the feature genes from DEICRGs, we trained two machine learning algorithms of lasso and SVM-RFE. The lasso regression is a machine learning algorithm involving a linear relationship assumption and an L1 regularization penalty. First, the lasso regression with the minimum binomial deviance was performed through 10-fold cross-validation. Genes with non-zero regression coefficients were selected for feature genes of DEICRGs. As a result, there were 14 genes included in the simplified lasso regularization model (Figures 6A,B; Table 3).

**TABLE 3:** Feature genes of lasso and SVM-RFE.

Machine learning algorithm	Feature gene
Lasso	CLCN5、FKBP1B、ASPH、MCOLN1、ANO3、TRPM3、ATP9A、ATP8A1、ATP11C、ATP9B、ATP4A、FXYD3、CAMK2G、and ATP6V0E1
SVM-RFE	CAMK2G、ATP6V0E1、ASPH、MCOLN1、ATP8A1、ATP4A、CLIC2、ATP9B、ATP11C、ANO3、ATP2C1、and WNK2

SVM-RFE, a powerful machine learning paradigm for classification, regression, and other machine learning tasks, has often been reported to outperform other machine learning classifiers (Cai et al., 2020). Then, the optimal feature gene extraction strategy was applied using the SVM-RFE machine learning method. When the number of feature genes reached 12, the model achieved the maximal accuracy of 94.9% and a minimum error of 5.1% simultaneously. Thus, these top 12 feature genes are considered optimal and used for further analyses (Figures 6C,D; Table 3).

### PPI network and hub gene analyses

**FIGURE 7:**
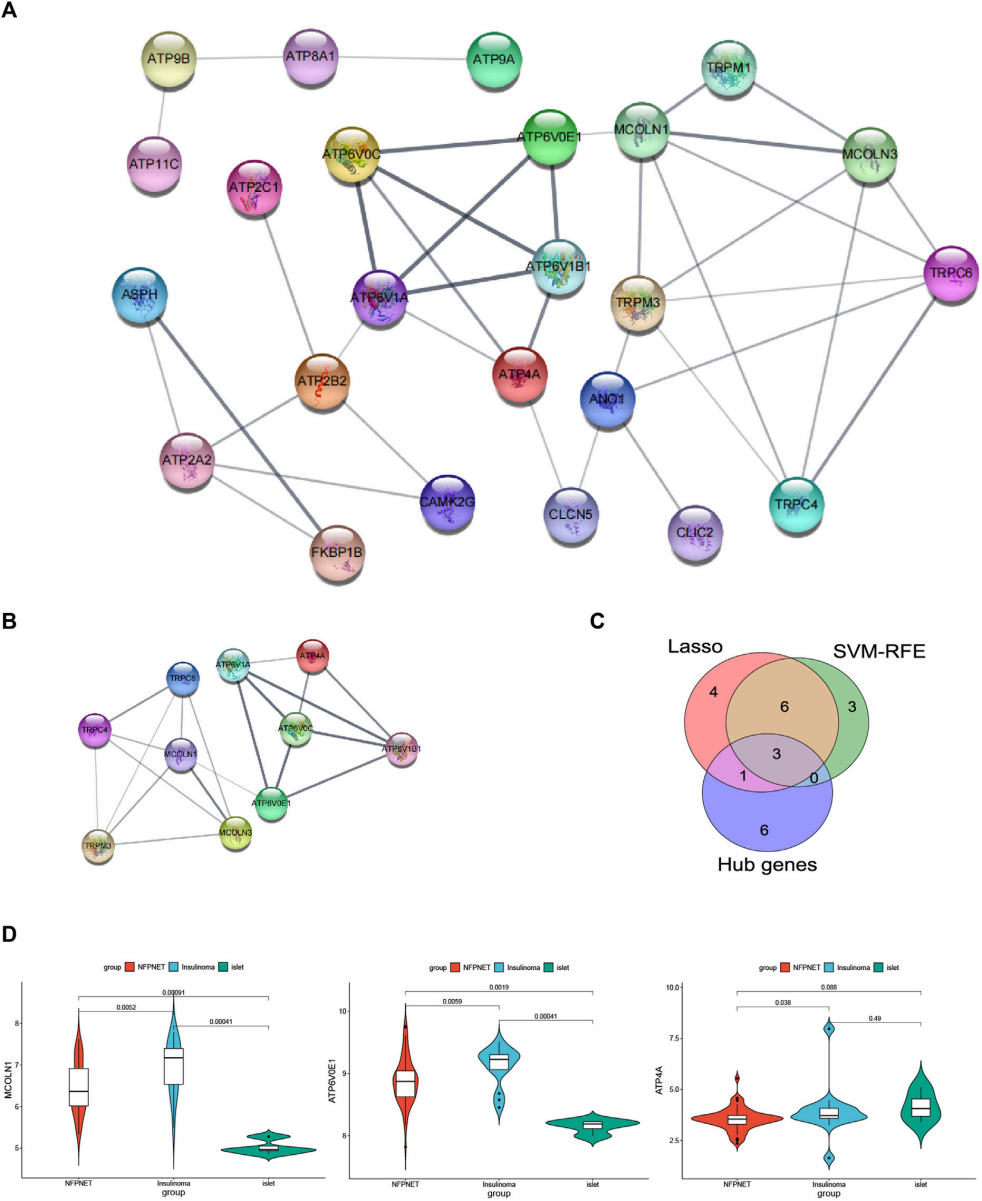
PPI network and recognition of target genes. **(A)** PPI network of the DEICRGs, each node represented a protein, each edge represented an association between two proteins, and the bigger sizes of the edge mean the higher MCC scores; **(B)** PPI network of the top 10 hub genes; **(C)** Venn diagram for recognizing target genes. **(D)** mRNA expression MCOLN1, ATP4A, and ATP6V0E1 in GSE73338 between NFPNET, insulinoma, and islet groups.

A PPI network of the DEICRGs was constructed to explore the connection within each protein, including 24 nodes and 41 edges. In this PPI network map, each node represented a protein, and simultaneously, each edge represented an association between two proteins (Figure 7A). Then, we used the cytoHubba to select the PPI network hub genes with the maximal clique centrality (MCC) method. Finally, we extracted the top 10 hub genes, which might play a possibly essential role in the PPI network (Figure 7B).

### Recognition of target genes

We took an intersection among the three key gene sets screened by the PPI network (hub genes), lasso model, and SVM-RFE model, resulting in three target genes (*ATP4A*, *MCOLN1*, and *ATP6V0E1*) (Figure 7C). Then, the details of three target genes’ expression between insulinoma, NFPNET, and normal islet groups are shown in Figure 7D.

### Construction and assessment of the nomogram model for insulinoma diagnosis

**FIGURE 8:**
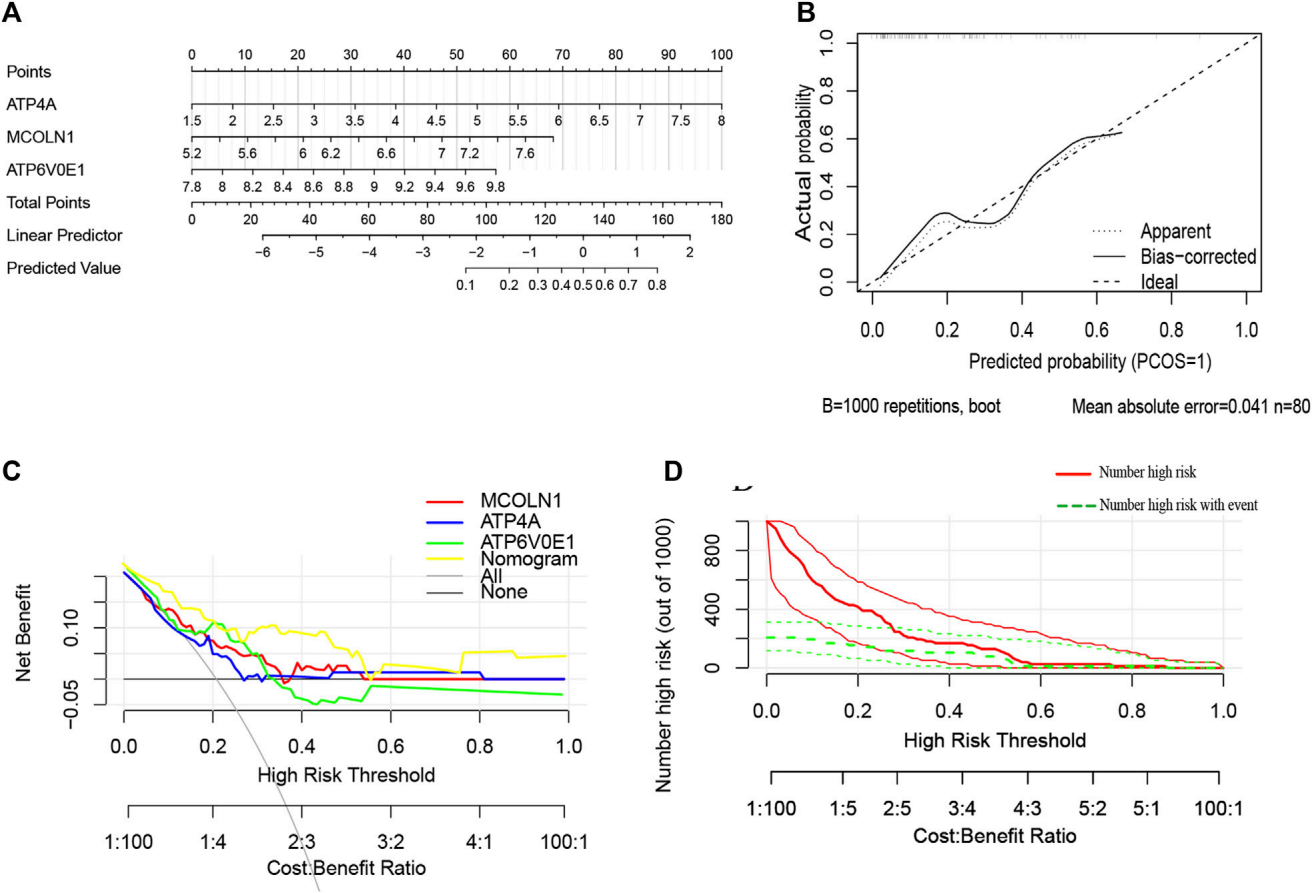
**(A)** Nomogram model predicting insulinoma based on three target genes. The nomogram is used by summing all points identified on the scale for each variable. The total points projected on the bottom scales indicate the probabilities of insulinoma; **(B)** calibration curves for the nomogram with the mean absolute error = 0.041; **(C)** DCA of the nomogram model and each target genes (“all” means diagnosis-all strategy; “none” means diagnosis-none strategy) and the nomogram model had a higher net benefit at a given high-risk threshold mostly; **(D)** CIC of the nomogram model.

Furthermore, a nomogram model was constructed based on the multivariate Cox analysis of three target genes via the R rms package (Figure 8A). Then, a calibration curve was used to evaluate the predictive power of the nomogram model. Then, a calibration curve was used to evaluate the predictive power of the nomogram model. The calibration curve indicated that the error between the actual probability and predicted probability of insulinoma is minimal, with a mean absolute error of 0.041, suggesting this nomogram model owns high accuracy in predicting insulinoma (Figure 8B). There are 17 insulinoma samples and 63 NFPNET samples in the GSE73338 dataset, with the insulinoma samples accounting for 21.25%. According to the previous research, functional PNETs (containing insulinomas, gastrinomas, glucagonomas, VIPomas, and somatostatinomas) represent less than 30% of all PNETs and are associated with particular clinical syndromes (Grozinsky-Glasberg et al., 2015). Decision curve analysis (DCA) indicated that the “nomogram” curve was higher than the “all”, “ATP4A”, “MCOLN1”, “ATP6V0E1”, and “none” curves within the high-risk threshold from 0 to 0.54. This suggests that the patients could benefit from the nomogram model, and the clinical benefit of the nomogram model was mainly higher than that of all of the other curves (Figure 8C). A CIC on the ground of the DCA curve was drawn to evaluate the nomogram model’s clinical effects visually. The “number high risk” curve was close to the “number high risk with event” curve at a high-risk threshold from 0.2 to 1, which indicated that the nomogram model has extraordinary predictive power (Figure 8D). These results, to some extent, also indicated that these three target genes might play a remarkable role in the process of insulinoma.

### ROC curve analysis

**FIGURE 9:**
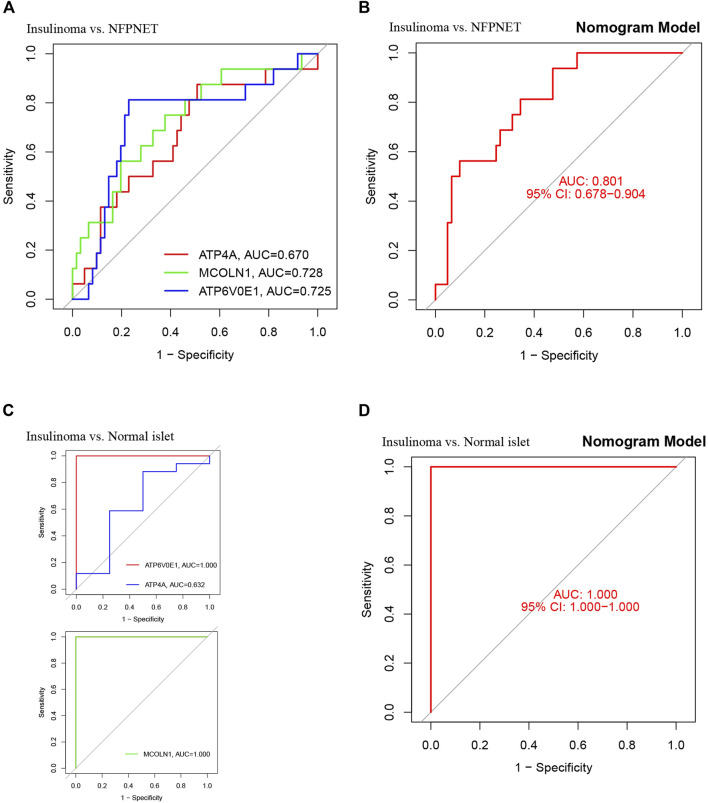
Validation of the nomogram model with the ROC curve. **(A)** ROC and AUC of each target gene between insulinoma and NFPNET groups; **(B)** ROC and AUC of the nomogram model between insulinoma and NFPNET groups; **(C)** ROC and AUC of each target gene between insulinoma and normal islet groups; **(D)** ROC and AUC of the nomogram between insulinoma and normal islet groups.

ROC curves with areas under the curve (AUC) are shown for investigating the diagnostic effectiveness of the nomogram model between insulinoma and NFPNET by using the identified three target genes (*ATP4A*, *MCOLN1*, and *ATP6V0E1*). ROC curve analyses revealed that the AUC was 0.728 for MCOLN1, 0.725 for ATP6V0E1, and 0.670 for ATP4A (Figure 9A). Additionally, the AUC was 0.801 [95% confidence interval (CI), 0.674–0.898] for the nomogram model by utilizing all three target genes simultaneously (Figure 9B). In contrast to normally differentiated β-cells, insulinoma cells remain continuously secreting insulin and proinsulin at low blood glucose (Guettier and Gorden, 2010). Therefore, the insulinoma and normal islet samples were applied to further verify the accuracy of this diagnostic nomogram model, with AUC tending to 1.0 (Figures 9C,D). These results suggested that these three target genes (*ATP4A*, *MCOLN1*, and *ATP6V0E1*) can serve as effective diagnostic biomarkers for distinguishing insulinoma from NFPNET and normal islets.

### Immune infiltration analysis

The heterogeneous tumor cells, immune cells, inflammatory cells, and intratumoral capillaries contribute to the tumor microenvironment heterogeneity, which is well known to modulate tumor growth and function (Bice et al., 2017). The intrapancreatic infiltration of immune cells and autoimmune attack with disorder immune homeostasis could destroy islet cells (Nelson et al., 2020). Then, we applied two disparate algorithms to identify the heterogeneous infiltration of immune cells in insulinoma.

**FIGURE 10:**
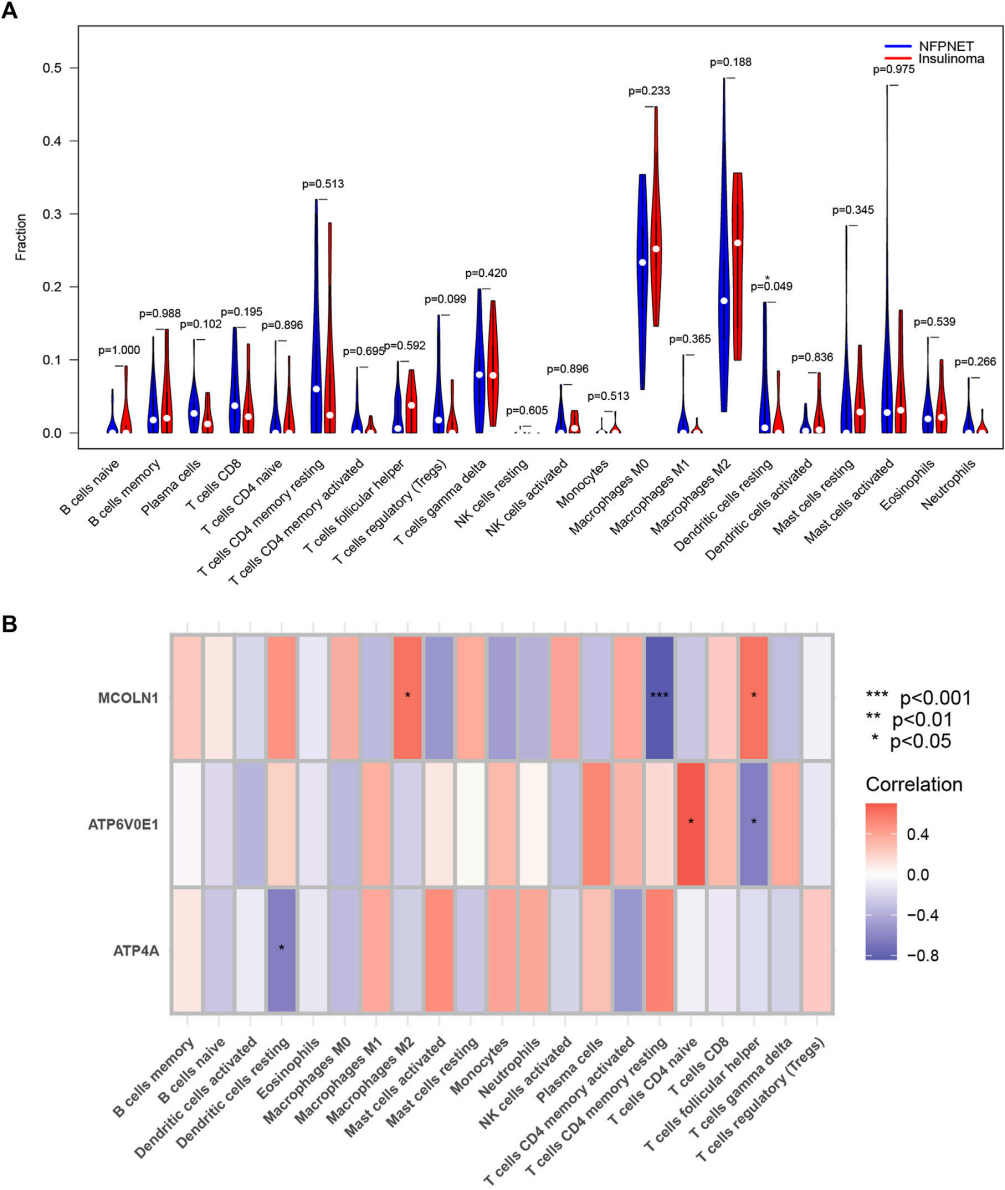
Immune infiltration analysis of the CIBERSORT algorithm. **(A)** Violin plot of the expression levels of 22 immunocyte subtypes in insulinoma and normal groups (* means *p* < 0.05); **(B)** Heatmap of the correlation coefficient between each target gene and each immunocyte subtype.

The CIBERSORT algorithm explored the dissimilarity of immune infiltration between insulinoma and NFPNET tissues. As shown in Figure 10A, insulinoma tissue had a lower ratio of dendritic cells resting within the 22 immune cells of the CIBERSORT algorithm. Next, the correlations between each subpopulation of immune cells and three target genes were displayed based on Pearson’s correlation coefficient. The outcomes revealed that M2 macrophages and follicular helper T cells were significantly positively associated with MCOLN1. On the contrary, the resting memory CD4 T cells were negatively associated with MCOLN1. ATP6V0E1 was positively related to CD4 naive T cells but negatively with follicular helper T cells. Moreover, resting dendritic cells were negatively correlated with ATP4A (Figure 10B).

**FIGURE 11:**
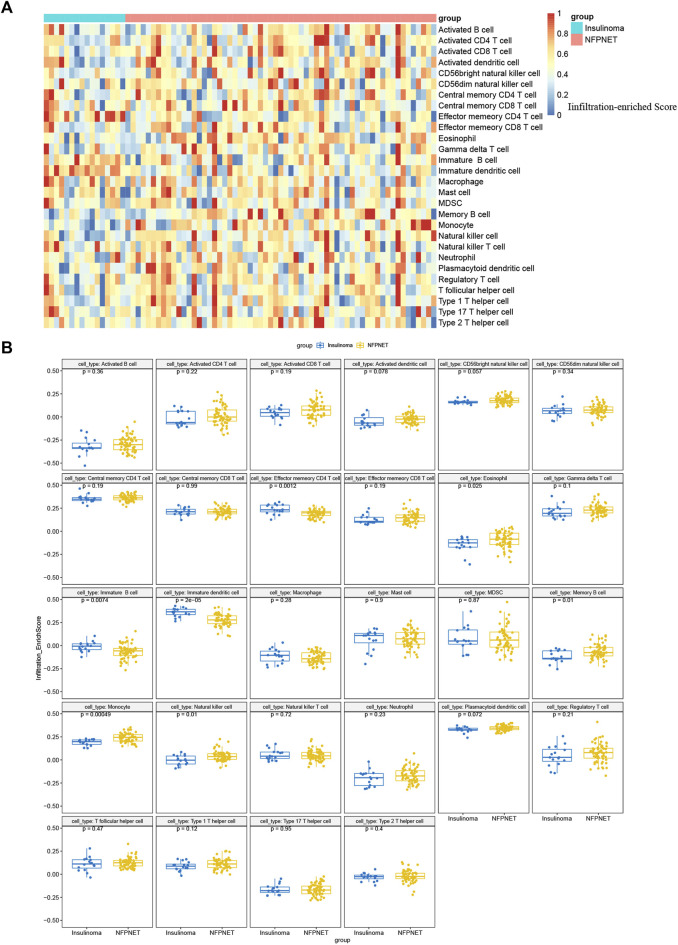
Single-sample gene set enrichment analysis for immune infiltration. **(A)** Heatmap based on the infiltration enrichment score of each sample; **(B)** boxplot of 28 immunocyte subtypes between insulinoma and NFPNET groups.

Furthermore, we used the ssGSEA to identify immune cell subtypes that are differentially represented in the tumor microenvironment among insulinoma and NFPNET tissues, while the immune-related gene set of 28 immunocyte subtypes was derived from 37 studies of microarray data (Charoentong et al., 2017). Then, the heatmap of the infiltration enrich scores based on each sample is shown in Figure 11A. As shown in Figure 11B, the memory CD4 T cells, immature B cells, and immature dendritic cells are significantly enriched in insulinoma tissue. However, the insulinoma group exhibited a lower infiltration enrichment score of eosinophils, memory B cells, monocytes, and natural killer cells.

### Single-gene GSEA and GSVA of target genes

Since *ATP4A*, *MCOLN1*, and *ATP6V0E1* might be pivotal in the ion channels for insulinoma and participate in regulating the secretion of various hormones, we selected these three target genes separately for further single-gene GSEA and GSVA analyses. The conclusions were primarily consistent with the previous results.

**FIGURE 12:**
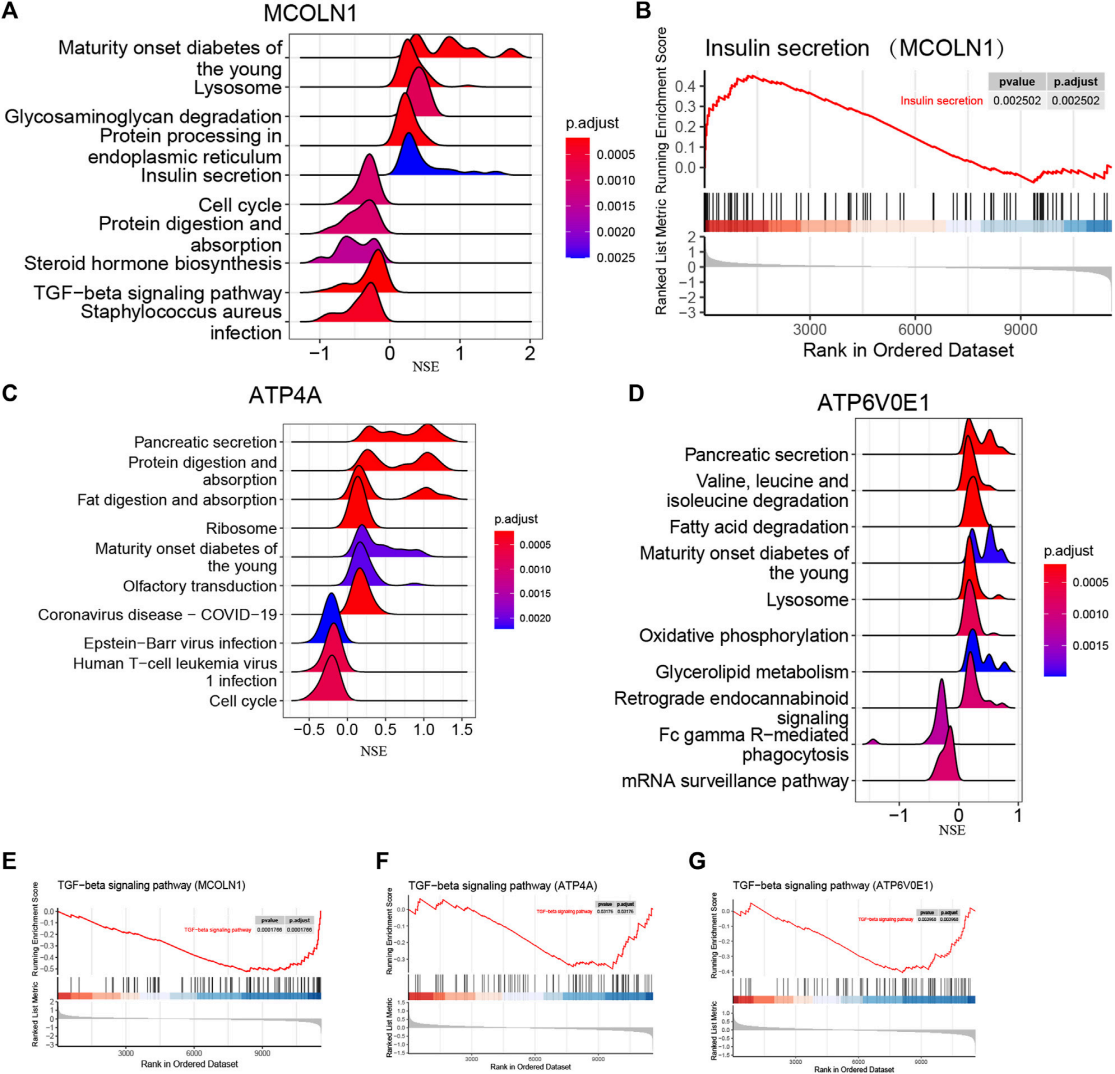
Single-gene GSEA of each target gene. **(A)** Top five upregulated and top five downregulated KEGG pathways ranked by the enrichment score from single-gene GSEA of MCOLN1; **(B)** insulin secretion pathway from single-gene GSEA of MCOLN1; **(C)** top five upregulated and top five downregulated KEGG pathways ranked by the enrichment score from single-gene GSEA of ATP4A; **(D)** top five upregulated and top five downregulated KEGG pathways ranked by the enrichment score from single-gene GSEA of ATP6V0E1; **(E–J)** insulin secretion pathway from single-gene GSEA of MCOLN1, ATP4A, and ATP6V0E1.

Based on the mRNA expression of each target gene, we separated all enrolled insulinoma and NFPNET samples into high-expression and low-expression groups. Then, the single-gene GSEA analyses were performed via KEGG pathways. As shown in Figures 12A,B, the activity of insulin secretion, lysosome, protein processing in the endoplasmic reticulum, and glycosaminoglycan degradation was upregulated in the MCOLN1 high-expression group. In comparison, the activity of the cell cycle, protein digestion and absorption, steroid hormone biosynthesis, and TGF-beta signaling pathway was downregulated. Furthermore, the upregulation of ATP4A (Figure 12C) and ATP6V0E1 (Figure 12D) is associated with a higher activity of the pancreatic secretion pathway. The lysosome, which may take part in managing insulin secretion and degradation of islet cells, is also correlated with ATP6V0E1 (Figure 12D).

Interestingly, the upregulation of three target genes was simultaneously related to the lower activity of the TGF-beta signaling pathway (Figures 12E–G). Taken together, we hypothesize that the upregulation of three target genes might be correlated with the upregulated activity of insulin secretion, lysosome, and pancreatic secretion. Correspondingly, the TGF-beta signaling pathway might play a vital role in insulinoma.

**FIGURE 13:**
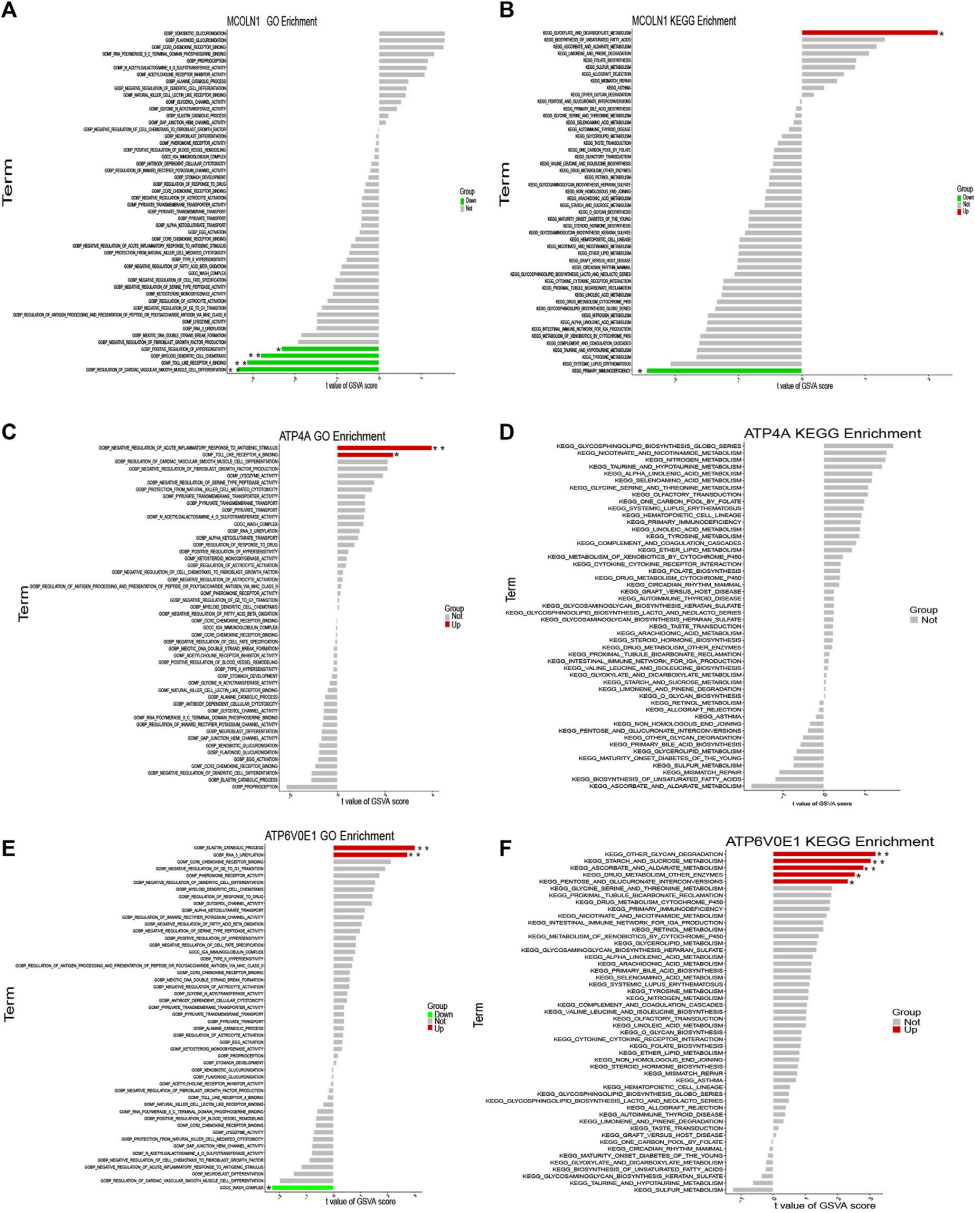
Single-gene GSVA of each target gene. **(A)** GSVA of MCOLN1 with the GO category; **(B)** GSVA of MCOLN1 with the KEGG pathway; **(C)** GSVA of ATP4A with the GO category; **(D)** GSVA of ATP4A with the KEGG pathway; **(E)** GSVA of ATP6V0E1 with the GO category; **(F)** GSVA of ATP6V0E1 with the KEGG pathway (*means *p* < 0.05; **means *p* < 0.01).

The GSVA analysis was performed via the GO function and KEGG pathways. The results of GO functions illustrated that the pathway activities of positive regulation of hypersensitivity, myeloid dendritic cell chemotaxis, toll-like receptor-4 binding, and regulation of cardiac vascular smooth muscle cell differentiation were significantly downregulated in the MCOLN1 high-expression groups (Figure 13A). In the KEGG pathway category, the glyoxylate and dicarboxylate metabolism pathway had a higher activity, but the primary immunodeficiency pathway was downregulated (Figure 13B). Similarly, the activities of various pathways were changed significantly with the perturbance of ATP4A and ATP6V0E1, and the details are provided in Figures 13C–F.

### Drug–gene interaction network

**FIGURE 14:**
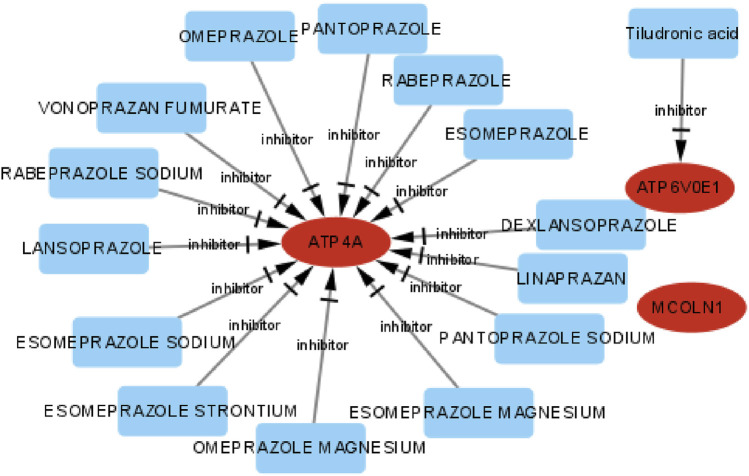
Drug–gene interaction network of target genes, the ellipse shape means target genes, the rectangle shape means drugs, and the red color means up-expression.

The DGIdb and DrugBank databases were applied to investigate the potential drug–gene interplay to distinguish existent or/and possibly related pharmaceutical materials. Exploring potential therapeutic drugs for targeting *ATP4A*, *MCOLN1*, and *ATP6V0E1* might offer a definite treatment approach to symptom improvement of insulinoma. The integrated drug–gene interaction network is displayed integrally (Figure 14). Finally, we distinguished 15 potential sanative drugs in total.

## Discussion

Insulinoma, featured with abnormally secreting insulin contrary to the NFPNET, can repeatedly lead to severe hypoglycemic neuro-glycogenic and sympathetic overexcitement symptoms (Metz and Jensen, 2008). The irreversibly severe impairment of the central nervous system function is the most significant hazard of insulinomas due to extraordinary hypoglycemia. Recently, the diagnosis of insulinoma mainly depends on clinical symptoms and the assessment of serum glucose, insulin, C-peptide, and β-hydroxybutyrate levels (Dauben et al., 2019; Giannis et al., 2020). The single-photon emission computed tomography (SPECT) is also applied to diagnose insulinoma by administering exendin-4 (a GLP-1 peptide analog), which has 95% sensitivity (Jansen et al., 2019). Unfortunately, the diagnosis of insulinoma is often delayed due to varied clinical presentations, low-specificity clinical diagnostic models, and the costly equipment of SPECT (Dauben et al., 2019; Giannis et al., 2020). Hence, early and accurate diagnosis and treatment are crucial for a better prognosis (Wu et al., 2017).

As previously described, ion channel-related genes might be involved in the peculiarity of insulinoma with unregulated insulin secretion, which is different from normal islet cells and NFPNET. In the present study, we first identified the DEGs between insulinoma and NFPNET samples. Then, a total of 650 DEGs were explored, and the results of enrichment analyses indicated that many ion channel-related pathways were involved. As expected, these findings are consistent with our prolepsis and promote us to further research. Furthermore, we identified 29 DEICRGs, which are mainly enriched in functions including cellular metal ion homeostasis and pathways such as the cAMP signaling pathway, calcium signaling pathway, and lysosome. It was reported that L-arginine can promote insulin secretion in β-cells through the GPRC6A stimulation of cAMP pathways (Pi et al., 2012), and incretin hormones increase the cAMP of β-cells (Kaihara et al., 2013). The upregulation of the cAMP concentration can activate protein kinase A and/or GEFII to accelerate insulin vesicle exocytosis (Zhao et al., 2010). Additionally, both the calcium channel and lysosome participate in the regulation of insulin secretion (Ning et al., 2008; Asadi and Dhanvantari, 2020). Therefore, we logically assume that the heterogeneous expression of ion channel-related genes between insulinoma and NFPNET may contribute to the heterogeneity ability of insulin secretion.

Machine learning algorithms outperform standard logistic regression in constructing precise classification and prediction models of diseases (Ross et al., 2016). An integrated algorithm of lasso regression and SVM-RFE was utilized to screen out the featured genes with the highest prediction accuracy. Then, three target genes (*ATP4A*, *MCOLN1*, and *ATP6V0E1*) were finally derived from the intersection of featured genes and hub genes. A nomogram model that exhibits extraordinary predictive efficacy was established and confirmed with the calibration curve, DCA, and CIC. The ROC curve was applied to evaluate the availability of the nomogram model with the 0.801 AUC (95% CI 0.674–0.898). Moreover, the insulinoma and normal islet samples were also used to verify the model’s accuracy, with the AUC tending to 1.0. In summary, these outcomes suggest that *ATP4A*, *MCOLN1*, and *ATP6V0E1* are crucially ion channel-related genes in insulinoma, and the diagnostic model based on these genes was highly efficient.

MCOLN1, located in the lysosome, is an unselective cation channel and plays an essential role in modulating various intracellular processes containing endocytosis, exocytosis, lysosomal adaptation, and autophagy (Qi et al., 2021). MCOLN1 provides a negative feedback accumulation of mTORC1 to prevent immoderate wastage of mTORC1 activity within the starvation and other stress states by regulating the Ca^2+^ flux of lysosomes and the mTORC1-dependent autophagy pathway (Sun et al., 2018). Autophagy, intra-lysosomal proinsulin degradation, and insulin secretory granule transport are strictly governed, which are essential to maintain insulin homeostasis (Fujitani and Watada, 2012; Li et al., 2022). Numerous reports have suggested that the activation of mTORC1 promotes protein translation and insulin synthesis, and triggers pancreatic β-cell proliferation and growth. The pancreatic β-cell-specific mTOR-knockout mice tend to have an abnormal mitochondrial function and reduced insulin secretion (Mori et al., 2009; Asahara et al., 2022). Since then, we speculate MCOLN1 might be involved in mediating insulin secretion by regulating the lysosomal function, autophagy, and mTORC1 activation. Then, we applied MCOLN1-specific single-gene GSEA to explore the critical mechanisms. Exhilaratingly, the up-expression of MCOLN1 is significantly positively associated with the activation of lysosome and insulin secretion, which corresponds with the literature and supports our hypothesis.

ATP6V0E1 belongs to the V-ATPase family and encodes the membrane-bound subunit e of V-ATPase, a multisubunit enzyme that mediates the acidification of eukaryotic intracellular organelles (Stransky et al., 2016). Lysosomal acidification mechanisms based on the V-ATPase and the counter ion transporter are critical for proper lysosome function (Mindell, 2012). Over-expressed ATP6V0E1 of macrophages partially rescued TcdB-triggered downregulation of lysosomal proton pump subunits and lysosome neutralization (Chan et al., 2022). Lysosomal acidity and autophagic flux of pancreatic β-cells are suppressed by exposure to persistent lipotoxicity. These can be reversed through lysosome acidification by delivering lactic and glycolic acid into lysosomes, resulting in restoring autophagic flux and insulin secretion of pancreatic β-cells (Zeng et al., 2019). Recently, the gastric proton pump H+/K + -ATPase, encoded by ATP4A, has been reported to affect pancreatic secretion (Wang et al., 2015). ATP4A promotes the exocytosis of insulin vesicles by triggering the closure of KATP channels and regulating the calcium influx through voltage-dependent calcium channels. The knock-down of ATP4A in the insulinoma cell line MIN6 led to decreased glucose-stimulated insulin secretion (Schallschmidt et al., 2018). In our study, single-gene GSEA indicated that both ATP6V0E1 and ATP4A are positively associated with the pancreatic secretion pathway. Meanwhile, ATP6V0E1 is also positively correlated with the lysosome. These outcomes are consistent with previous literature reviews and show a regulatory role of ATP6V0E1 and ATP4A in insulin secretion.

Interestingly, the upregulated *MCOLN1*, *ATP6V0E1*, and *ATP4A* were conformably associated with decreased activity of the TGF-beta signaling pathway. It has been suggested that the inhibitor of TGF-beta receptor I can significantly stimulate C-peptide secretion and promote human islet β-cell proliferation by suppressing the activity of the TGF-beta signaling pathway (Xiao et al., 2016). Z. Zi reported that the degradation of TGF-beta in lysosomes is important for suppressing TGF-beta signaling (Zi et al., 2012). In summary, MCOLN1, ATP6V0E1, and ATP4A might enhance the insulin secretion of insulinomas by participating in regulating the function and TGF-beta signaling pathway.

In the CIBERSORT algorithm for immune infiltration analysis, we found that the fraction score of resting dendritic cells was significantly lower in the insulinoma group with a trend of increased M2 macrophages. Moreover, MCOLN1 was positively correlated with M2 macrophages, whereas ATP4A was negatively correlated with resting dendritic cells. Additionally, compared with NFPNET, the results of ssGSEA revealed that the infiltration-enriched scores of natural killer (NK) cells were significantly lower in insulinoma tissues, and insulinoma tissue seemed to have a tendency to have a lower infiltration-enriched score of activated dendritic cells. At present, there is no direct research evidence for the relationship between insulinoma and different types of dendritic cells. Mclaughlin R.J suggested that dendritic cells induce the posttranslational modification of islet autoantigens and trigger the autoimmune injury to islet βcells (McLaughlin et al., 2016). Furthermore, dendritic cells can efficiently phagocytize enterovirus-infected Min6 mouse insulinoma cells *in vitro* (Schulte et al., 2010). Therefore, we hypothesized that insulinoma tissue might have a lower infiltration of dendritic cells to escape immune damage, which is an interesting result that needs to be confirmed by further studies. It is well known that M2 macrophages perform an anti-inflammatory, prooncogenic, and immune-suppressive role within the tumor immune microenvironment (Pathria et al., 2019). Recent reports suggest that M2 macrophages can promote the immune escape of tumors by repressing the anti-tumor activity of cytotoxic CD8^+^ T cells (Peranzoni et al., 2018). Group VIA Ca^2+^-independent phospholipase A2 (iPLA2β) plays an impellent role in islet β-cell programmed cell death (Lei et al., 2010), whereas M2 macrophages accelerate the diminution of iPLA2β (Ashley et al., 2016). NK cells, a classic subtype of cytotoxic lymphocyte for innate immunity, take various approaches to kill carcinoma cells straightly (Kalluri, 2016). Consequently, insulinoma might exhibit a lower level of antineoplastic immune injury. The identified three target genes might partially contribute to the heterogeneous immune microenvironments of insulinomas and depress the immune attack in consideration of the correlation of these genes with immunocytes.

Our study also had some limitations. Significantly, the datasets and samples of mRNA expression profiles of insulinoma are few within the GEO database due to the low incidence and difficulty of diagnosis, which need to be verified in other datasets in future. However, it is very fortunate that the diagnostic model was also influential in distinguishing insulinoma and normal islets, which would partially enhance our model’s credibility. In addition, in future, further experiments need to be carried out to corroborate our outcomes. Integrating these target genes with other clinical diagnostic models and targeting them might also be considerable and valuable.

## Conclusion

We identified three target ion channel-related genes (*MCOLN1*, *ATP6V0E1*, and *ATP4A*) through integrated bioinformatics and two machine learning algorithms. We also constructed an efficiently predictable diagnosis model for identifying insulinoma, which might offer a novel approach for diagnosing insulinoma in clinical practice.

## Data Availability

The original contributions presented in the study are included in the article/Supplementary Material; further inquiries can be directed to the corresponding authors.
