# Supplementary figures and images for: Identifying target ion channel-related genes to construct a diagnosis model for insulinoma
Source: Front Genet. 2023 Sep 12;14:1181307. doi: 10.3389/fgene.2023.1181307 (PMC10523017; doi:10.3389/fgene.2023.1181307)

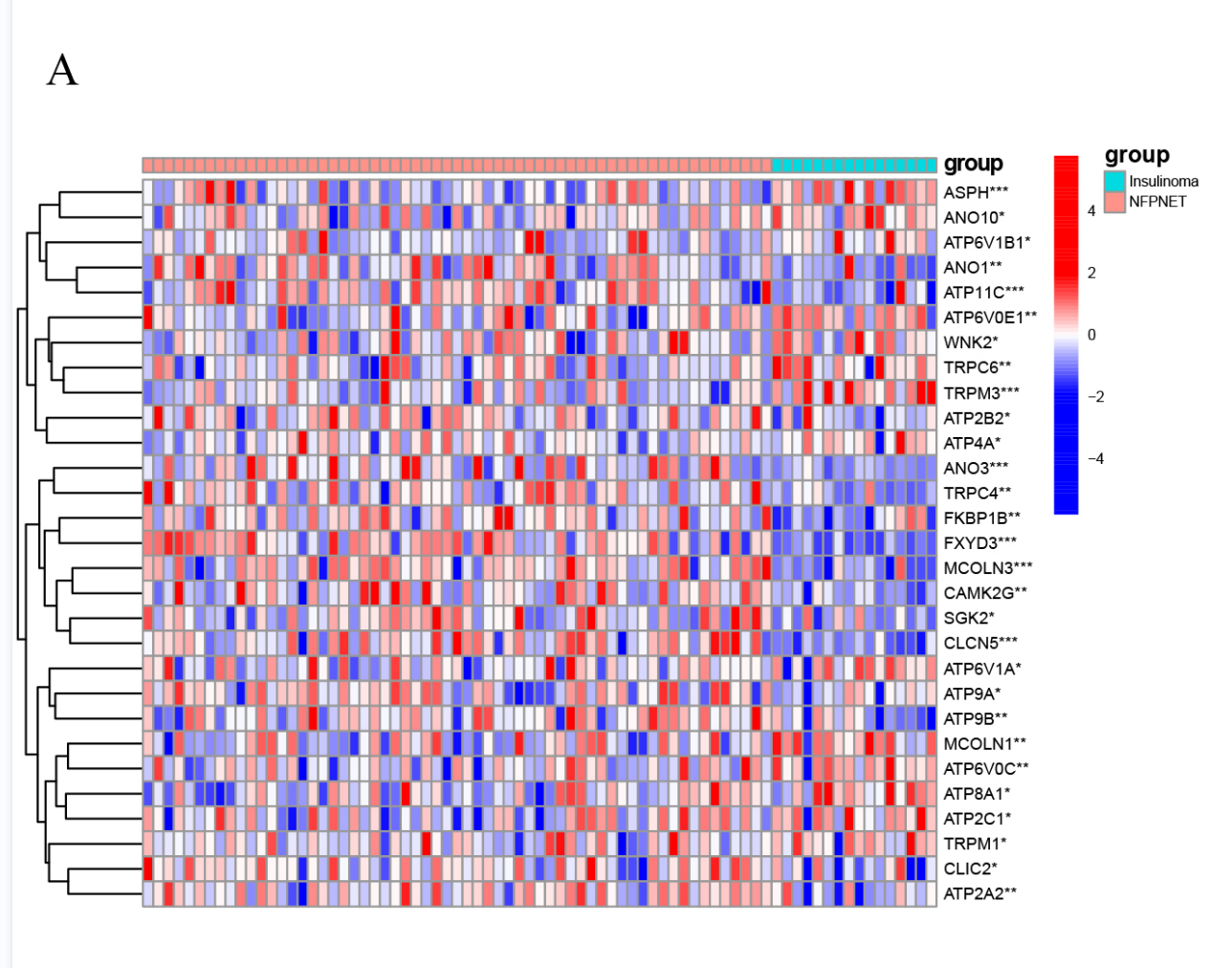

Supplement: Supplementary file 4 [file Image15.png]
